# Environmental enrichment and visceral inflammation regulate stress-induced c-Fos and NPY expression within the dentate gyrus

**DOI:** 10.1186/2050-6511-13-S1-A47

**Published:** 2012-09-17

**Authors:** Florian Reichmann, Evelin Painsipp, Peter Holzer

**Affiliations:** 1 Research Unit Translational Neurogastroenterology, Institute of Experimental and Clinical Pharmacology, Medical University of Graz, 8010 Graz, Austria

## Background

The dentate gyrus, a part of the hippocampal formation, is an important brain region regulating the central response to stress. Given that stress resilience depends on genetics and adaptive processes within the brain, the question arises as to whether the acute stress response is modified by chronic environmental or pathological conditions. Therefore we investigated, on the one hand, whether environmental enrichment (EE), a housing condition suggested to promote stress resilience, alters the acute stress response. On the other hand, we assessed whether visceral inflammation, known to be exacerbated by stress, also has an impact on the central stress response.

## Methods

Mice were housed in a standard or enriched environment for 10 weeks. During week 10, mice were treated either with iodoacetamide (IAA, 0.1% in drinking water) to induce gastritis or dextran sulfate sodium (DSS, 2% in drinking water) to induce colitis; control mice received plain water. At the end of the treatment period the mice were exposed to water avoidance stress, a psychological stressor, for 30 min. Two hours later post-stress c-Fos expression was measured immunohistochemically and post-stress neuropeptide Y (NPY) expression was investigated in the dentate gyrus by quantitative *in situ* hybridization.

## Results

Two-way ANOVA revealed that EE increased post-stress c-Fos expression within the dentate gyrus in control (p = 0.003) and gastritis (p = 0.027) animals but not in colitis animals (n = 6–8). Furthermore, an inhibitory effect of gastritis and colitis on stress-induced c-Fos expression was observed in mice under EE. NPY expression per neuron was altered by both EE (p = 0.002) and visceral inflammation (p = 0.001). Specifically, post-stress NPY expression was higher in mice under EE (254.1 ± 17.3 grains/neuron) compared to standard-housed mice (191.7 ± 13.7 grains/neuron), independently of treatment conditions, and NPY expression was higher in gastritis mice (275.1 ± 17.5 grains/neuron) compared to control animals (204.2 ± 22.2 grains/neuron), independently of housing conditions.

## Conclusions

These results indicate that processing within the dentate gyrus of an acute stress exposure is distinctly altered by EE and gastrointestinal inflammation. EE facilitates, whereas gastritis and colitis blunt the stress-induced neuronal activation visualized by c-Fos. The EE-induced increase in the stress response is paralleled by an increase in the expression of NPY in the dentate gyrus. This shows that NPY is not only involved in the stress response but also participates in EE-evoked neuronal plasticity.

